# Note on Tremors

**Published:** 1918-10

**Authors:** 


***Note on Tremors***. M. Henry MEIGE et Mme A. BENISTY, *Société de Neurologie de Paris*, Jan. 1918.

The question of tremors is a very difficult one. Very little can be told of their clinical differentiation, pathological anatomy, or pathogeny; the best and every case is a medico-legal problem. Clinical analysis is a means of determining differential characteristics.

The cases of a number of soldiers affected with tremor, — from the service of Prof. Pierre Marie of the Salpêtriere — were presented.

(1) Parkinson's type, (2) Neuropathic tremor, (3) Tremor persisting during sleep, (4) Head tumor, (5) Tremor with camptocormia, (6) Emotional tremor, (7) Constitutional tremor.... In every case, the form, location, and evolution of the tremor must be studied. The form gives useful indications as to its nature. The slow type belongs to the organic form (Parkinsonian); so does the very rapid type (general paralysis, Basedow's, intoxications). The medium rhythm type is mostly characteristic of neuropathic conditions. This classification is relative; it is much more important to note the regularity or irregularity of the tremor. A tremor always remaining similar to it is most often of organic origin. An irregular type, on the contrary, is neuropathic, emotional, or constitutional.

*Location.* Very often it is hard to localize the exact seat of the trouble. Where tremor distinctly affects the fingers, it strongly suggests an organic origin (Parkinson's). The proximal types are generally of neuropathic origin, and the distant types are organic ones. The tremors affecting the head only (*hemitremblements*) indicate an organic origin, while those affecting the inferior limbs only are scarce, and generally neuropathic. The synchronism of the oscillations is very important; it is always constant in Parkinson's disease. Tremor must be observed during movement and at rest. The types presenting paroxysms are emotional.

*Evolution.* — (1) *The progressive type* : extending from one limb or part of it to other limbs on one or both sides (Parkinson's). (2) *Regressive type* : general at first; has tendency to regress to one side or one limb (neuropathic ore motional). (3) *Migratory* : changing from one place to another (neuropathic). It is also very desirable to find out if the tremor persists during sleep. The morphologic subject is Parkinson's; muscles never appear to be relaxed. The speed amplitude and precision of movement (as in writing) are also noted. Palpitation will show the waxy flexibility in Parkinsonian types, and an intermittent (cog wheel) resistance in neuropathic tremors. The examination of reflexes is not easy. Mental examination must be made in every case.

The motor trouble itself is only one of the dystonic symptoms which should be studied as a whole.

